# Cognitive Deficits and Associated ERP N400 Abnormalities in FXTAS With Parkinsonism

**DOI:** 10.3389/fgene.2018.00327

**Published:** 2018-09-18

**Authors:** Xiao-Hong Wang, Jin-Chen Yang, Robert Soohoo, Devyn Cotter, Mei Yuan, Jiangyi Xia, Shuja Yaqub, Jesse Doty, Yu-Qiong Niu, Flora Tassone, Randi Hagerman, Lin Zhang, John Olichney

**Affiliations:** ^1^Department of Neurology, Dalian Municipal Central Hospital, Dalian, China; ^2^Department of Neurology, School of Medicine, University of California, Davis, Davis, CA, United States; ^3^Center for Mind and Brain, University of California, Davis, Davis, CA, United States; ^4^Department of Neurology, The Second Affiliated Hospital, University of South China, Hengyang, China; ^5^MIND Institute, School of Medicine, University of California, Davis, Davis, CA, United States; ^6^Department of Biochemistry and Molecular Medicine, University of California, Davis, Davis, CA, United States; ^7^Department of Pediatrics, School of Medicine, University of California, Davis, Davis, CA, United States

**Keywords:** FMR1 premutation, cognition, event-related potential (ERP), parkinsonism, semantic processing

## Abstract

**Objective:** To examine cognitive deficits and associated brain activity in fragile X-associated tremor/ataxia syndrome (FXTAS) patients with parkinsonism (FXTp+), in relation to FXTAS patients without parkinsonism (FXTp-), and normal elderly controls (NC).

**Methods:** Retrospective reviews were performed in 65 FXTAS patients who participated in the event-related brain potential (ERP) study and also had either a videotaped neurological examination or a neurological examination for extrapyramidal signs. Parkinsonism was defined as having bradykinesia with at least one of the following: rest tremor, postural instability, hypermyotonia, or rigidity. Eleven FXTp+ patients were identified and compared to 11 matched FXTp- and 11 NC. Main ERP measures included the N400 congruity effect, N400 repetition effect, and the late positive component (LPC) repetition effect.

**Results:** When compared with FXTp- and NC, the FXTp+ group showed more severe deficits in executive function, cued-recall, recognition memory, along with a significantly reduced N400 repetition effect (thought to index semantic processing and verbal learning/memory) which was correlated with poorer verbal memory. Across all patients, *FMR1* mRNA levels were inversely correlated with delayed recall on the California Verbal Learning Test (CVLT).

**Interpretation:** The findings of more prominent executive dysfunction and verbal learning/memory deficits in FXTp+ than FXTp- are consistent with findings in Parkinson’s disease (PD), and may indicate that concomitant and/or synergistic pathogenetic mechanisms associated with PD play a role in FXTAS. These results have implications not only for understanding the cognitive impairments associated with the parkinsonism subtype of FXTAS, but also for the development of new interventions for these patients.

## Introduction

Fragile X-associated tremor/ataxia syndrome (FXTAS) is a late-onset neurodegenerative disorder that primarily affects carriers of premutation CGG repeat expansions (range: 55–200) in the fragile X mental retardation 1 (*FMR1*) gene (Hagerman et al., 2001). The *FMR1* premutation produces elevated *FMR1* mRNA levels and leads to RNA gain-of-function toxicity, thought to be a fundamental mechanism of pathogenesis in FXTAS (Tassone et al., 2000; Hagerman and Hagerman, 2016). Neuropathological studies in FXTAS have revealed intranuclear *FMR1* mRNA-containing inclusions in neurons and astrocytes throughout the central nervous system (Greco et al., 2002). Radiological anomalies in FXTAS include brain atrophy and white matter diseases frequently found in the middle cerebellar peduncles (the MCP sign), the periventricular area, and the corpus callosum. Phenotypic manifestations of FXTAS are characterized by intention tremor, cerebellar ataxia, and cognitive decline. Other common clinical presentations of FXTAS include parkinsonian features (Hall et al., 2009) (a minor diagnostic criterion for FXTAS) and polyneuropathy (Hagerman and Hagerman, 2013, 2016). Patients with FXTAS have been found to have increased prevalence of parkinsonian features including bradykinesia (57%), rest tremor (26%), and postural instability (42%) (Niu et al., 2014). In that study, bradykinesia scores in those FXTAS patients were significantly correlated with both *FMR1* mRNA level and FXTAS clinical stage. Some of the patients with concomitant diagnoses of FXTAS and Parkinson’s disease (PD) or dual pathologies of the two disorders showed clinical benefits from dopaminergic treatment, whereas some displayed dopamine deficiency on SPECT imaging with an absence of response to L-dopa (De Pablo-Fernandez et al., 2015).

Cognitive deficits in FXTAS are well-established as impairments in executive function, short-term memory, and visuospatial processing. Milder cognitive difficulties in these same domains have been documented in premutation carriers asymptomatic of FXTAS (Grigsby et al., 2014; Wheeler et al., 2014). For 11 PD patients carrying GZ and lower end premutation alleles (41–85 repeats), Trost et al. (2014) reported significant correlations of white matter hyperintensities on MRI with both CGG repeats and neuropsychological test scores of general ability, working memory, and verbal learning. However, cognitive profiles of a subtype of FXTAS with parkinsonism (defined as having bradykinesia and at least one other main parkinsonian feature) remains largely unknown. As mentioned previously, Niu et al. (2014) performed a retrospective study using videotaped neurological exam data in a cohort of 38 patients with FXTAS, but their analyses were focused on parkinsonian motor features among these patients. In the current study, neuropsychological tests and event-related brain potentials (ERPs) were employed to examine cognitive functions and associated electrophysiological indexes in FXTAS patients with parkinsonism, in relation to FXTAS patients without parkinsonism and age matched healthy controls.

## Patients and Methods

### Participants

Retrospective reviews of data from 65 FXTAS patients who participated in the ERP study and also have either a videotaped neurological examination or a neurological examination performed by J.M.O for extrapyramidal signs. Parkinsonism was defined as bradykinesia with at least one of the following: rest tremor, postural instability, or hypermyotonia (in the videotaped cases), or rigidity (as assessed in neurological exam by J.M.O) (Hughes et al., 1992). Participants had provided written informed consent for a study protocol approved by the University of California Davis Institutional Review Board. There were 14 FXTAS patients with parkinsonism (FXTp+) identified by neurologists X-H.W. and J.M.O., two of which had unusable EEG data due to excessive artifacts and one of which was a non-native English speaker and thus did not complete the ERP word repetition experiment. Therefore, 11 FXTAS patients were included in the final sample of FXTp+. 11 FXTAS patients without parkinsonian symptoms (FXTp-) were selected to match FXTp+ patients in a one-on-one fashion based on demographics (age, education) and MMSE score. In addition 11 demographically matched normal controls (NC) were also analyzed.

*FMR1* CGG repeat length and *FMR1* mRNA level were quantified following procedures described elsewhere (Tassone et al., 2000, 2008). All patients had received a FXTAS diagnosis according to published criteria for probable or possible FXTAS (Jacquemont et al., 2003). Demographics and genetic measures are summarized in **Table 1**.

**Table 1:** Demographics and genetic-molecular measures (Mean ± *SD*) NC.

	NC (*n* = 11)	FXTp- (*n* = 11)	FXTp+ (*n* = 11)	*P*
Age	63.7 ± 7.7	63.7 ± 9.2	62.2 ± 7.7	0.88
Education (years)	15.72 ± 1.85	14.55 ± 3.05	15.09 ± 2.88	0.58
Females (N)	5	5	5	
Left-handed (N)	1	1	1	
FXTAS stage	–	3.10 ± 0.88	3.27 ± 0.65	0.80^#^
CGG repeats	32.43 ± 7.83	96.18 ± 33.57	84.75 ± 14.65	0.51^#^
*FMR1* mRNA	1.42 ± 0.38	2.99 ± 0.62	3.05 ± 0.52	0.98^#^
(*n* = 20)	(*n* = 6)	(*n* = 7)	(*n* = 7)	


*NC, normal controls; FXTp-, FXTAS patients without parkinsonism; FXTp+, FXTAS patients with parkinsonism. FXTAS disease stages were based on the functioning levels as defined in Jacquemont et al. (2003). ^#^Indicates p-values from comparisons between two FXTAS subgroups.*

### Neuropsychological Testing

Each subject underwent extensive neuropsychological evaluations. The Mini-mental state examination (MMSE) (Folstein et al., 1975; Folstein, 1990) was used as a measure of global cognitive abilities. Executive function was evaluated using the Stroop Color and Word Test (Stroop) (Spreen, 1998), Behavioral Dyscontrol Scale-2 (BDS-2) (Grigsby, 1996) and controlled oral word association test (COWAT) (Desrosiers and Kavanagh, 1987). The color-word component of the Stroop Test is a measure of cognitive inhibition. The BDS-2 provides a valid and reliable measure of behavioral self-regulation involving intentional control of voluntary motor behavior. The COWAT is a measure of verbal fluency, widely considered to be a component of executive function. Verbal memory was assessed with the California Verbal Learning Test (CVLT) (Delis et al., 1987).

### EEG/ERP Experiment

Thirty-two-channel EEG recordings were obtained during a cross-modal category judgment/word repetition paradigm in which semantically congruous category-exemplar (e.g., “a type of meat” – “chicken”) or incongruous nouns (e.g., “a part of the day” – “sapling”) pairs were presented and then repeated ∼10–140 s later. Category statements were read aloud, each followed ∼1 s later by a visually presented target word presented for 300 ms. Subjects were instructed to wait 3 s following the appearance of the target, then say the perceived word aloud, and use “yes/no” to indicate whether or not it was an exemplar of the defined category.

Participants were assigned to one of the three counterbalanced stimulus lists, which consist of 36 congruous category statement-target pairs presented once, 36 presented twice, 36 presented three times, and the equal numbers of incongruous stimuli in the same repetition patterns. In the total of 432 trials, 50% of the stimuli were congruous while 50% were incongruous, and 50% were new, while 50% were repeats. A repeated target word always followed the same category statement as in the first presentation. For stimuli repeated once, the lag between the first and second presentations was 10–40 s. For stimuli repeated twice, the lag for both second and third presentations was ∼100–140 s after the first presentation.

The EEG session lasted slightly over 20 min. More details of the ERP word repetition paradigm in FXTAS, including the EEG montage used, were described in previously published studies (Olichney et al., 2010; Yang et al., 2014b).

After the EEG recordings were completed, three unanticipated paper and pencil memory tests (free recall, cued recall, and then a multiple-choice recognition questionnaire) for the experimental stimuli used in the ERP study were administered.

### Data Analysis

EEG trials contaminated by eye blinks or movements, excessive muscle activity or amplifier blocking were rejected. By averaging artifact-free epochs of each condition, separate ERP waveforms were obtained for new congruous and incongruous target words, as well as for repeated congruous and incongruous target words. Mean ERP amplitude, peak amplitude and peak latency of N400 congruity effect (new congruous vs. incongruous words), N400 repetition effect (new vs. old incongruous words), and late positive component (LPC) repetition effect (new vs. old congruous words) were quantified. Repeated-measures ANOVAs were performed on each of the three main ERP measures from 24 scalp electrodes (excluding Fp1 and Fp2 because of their vulnerability to muscle activity; excluding POz and PO7 because of large electrode artifacts for two FXTAS patients). The Greenhouse-Geisser correction was used whenever the assumption of sphericity was violated. *Post hoc* tests were followed when warranted and Tukey’s multiple comparison test was used. *P-*values of < 0.05 were considered as significant for group comparisons.

Linear correlations between ERP measures, molecular/genetic measures, neuropsychological test scores, and subsequent memory scores for ERP experimental stimuli were examined. Pearson correlations were considered significant if *p* ≤ 0.05.

## Results

### Genetic and Molecular Testing

As expected, in all 22 patients with FXTAS, CGG repeats were significantly expanded [*F*_(2,27)_ = 17.07, *p* < 0.0001] and *FMR1* mRNA levels were significantly elevated [*t*_(1,18)_ = 41.96, *p* < 0.0001] when compared to normal controls. However, no significant differences on *FMR1* CGG repeats and mRNA levels were found between patients with and without parkinsonism (**Table 1**).

### Behavioral Results

**Table 2** summarizes neuropsychological test scores for the three groups. Compared to normal controls, the combined patient group of FXTp+ and FXTp- demonstrated poorer performance on global cognition (MMSE, *p* = 0.02), executive function (BDS-2, *p* = 0.02), inhibition (Stroop, *p* = 0.02), and verbal learning/acquisition (CVLT list A trials 1–5, *p* = 0.048). In contrast to FXTp-, the FXTp+ group was found to have more severe deficits in executive function as measured by BDS-2 and COWAT (*p*’s = 0.01), as well as a poorer memory for experimental stimuli (cued-recall: *p* = 0.03; multiple-choice: *p* = 0.05).

**Table 2:** Neuropsychological test scores compare (Mean ± SD).

	NC (*n* = 11)	^∗^*P*	FXTp- (*n* = 11)	FXTp+ (*n* = 11)	^#^*P*
**Global abilities**					
MMSE	28.55 ± 1.04	**0.02**	27.45 ± 1.37	27.00 ± 2.00	0.54
**Executive function**					
COWAT	44.11 ± 10.5	0.33	46.00 ± 16.33	28.33 ± 11.86	**0.01**
BDS-2	19.75 ± 4.43	**0.02**	17.30 ± 3.89	12.22 ± 3.73	**0.01**
Stroop	54.85 ± 12.1	**0.02**	47.00 ± 6.16	37.89 ± 12.49	0.08
**Verbal memory**					
CVLT list A, trials 1–5	52.63 ± 7.74	**0.048**	46.73 ± 12.84	36.67 ± 12.21	0.09
CVLT short delay free recall	10.63 ± 2.97	0.18	9.36 ± 3.80	7.56 ± 3.84	0.31
CVLT long delay free recall	10.75 ± 2.76	0.09	9.09 ± 4.28	6.66 ± 3.61	0.19
CVLT short delay cue-recall	11.5 ± 2.51	0.13	9.73 ± 3.23	9.33 ± 3.28	0.79
CVLT long delay cue-recall	12.00 ± 2.39	0.11	10.09 ± 3.81	9.22 ± 3.60	0.61
CVLT long delay discriminability	95.76 ± 2.82	0.08	85.56 ± 15.60	89.88 ± 8.25	0.46
**Subsequent memory (for ERP experimental stimuli)**					
Free recall	19.55 ± 11.83	0.07	12.45 ± 8.72	11.89 ± 11.47	0.90
Cued-recall	18.00 ± 3.16	0.08	17.27 ± 1.79	13.70 ± 4.69	**0.03**
Multiple choice	19.50 ± 1.58	0.46	20.18 ± 1.94	16.50 ± 5.50	**0.05**


*BDS-2, Behavioral Dyscontrol Scale-2; COWAT, Controlled Oral Word Association Test; CVLT, California Verbal Learning Test.^∗^P, NC vs. all FXTAS patients; ^#^P, FXTp*-*vs. FXTp+ subgroups. *P*’s < 0.05 are bolded above.*

### ERP Results

#### Repetition of Incongruous Words

**FIGURE 1:**
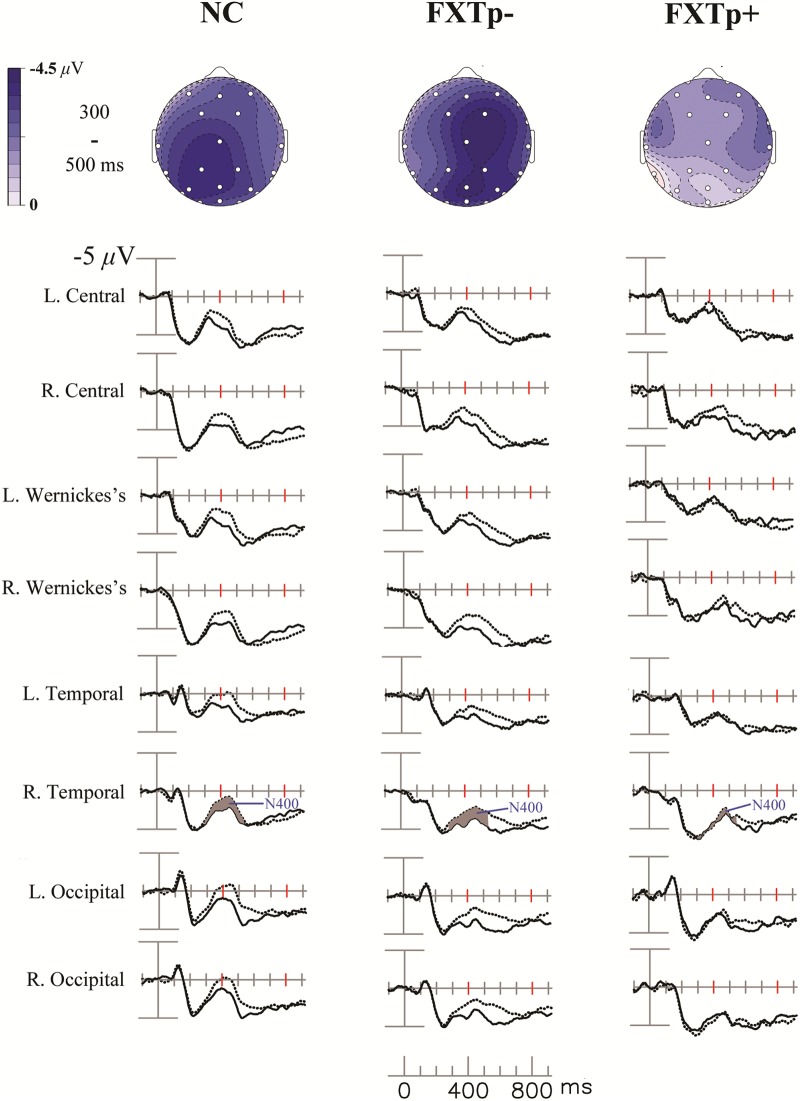
The N400 repetition effect. Upper: topographical maps of the N400 repetition effect (new–old incongruous words). Lower: grand average ERPs to initial (dotted line) and repeated (solid line) presentations of incongruous words, at eight selected temporal-posterior electrodes.

The grand-average ERPs and topography maps in **Figure 1** present the N400 word repetition effect (the difference between ERPs to initial and repeated presentations of incongruous target words).

Repeated measures ANOVA of the N400 mean amplitude between 300 and 550 ms after the onset of target words revealed a significant main effect of *repetition* (*F* = 29.64, *p* < 0.0001, η^2^= 0.5), confirming the presence of N400 repetition effect across all participants. ANOVA also found a significant interaction between *electrode* and *group* (*F* = 2.84, *p* = 0.019), and a marginally significant interaction between *repetition* and *group* (*F* = 3.26, *p* = 0.052). Further analysis focusing on a lateral-posterior (where the N400 repetition effect is most reliably obtained in normal subjects) cluster of 12 electrodes (41L/41R, WL/WR, CP1/CP2, T5/T6, P3/4, O1/O2) showed a significant interaction between *repetition* and *group* (*F* = 4.39, *p* = 0.02, η^2^= 0.23). *Post hoc* group comparisons indicated that FXTp+ group has significantly reduced N400 repetition effect amplitude (Mean = -0.26 μV, *SD* = 1.53) compared to both normal controls (Mean = -1.47 μV, *SD* = 1.27, *p* = 0.036) and FXTp- group (Mean = -1.43 μV, *SD* = 1.24, *p* = 0.044). No significant group difference was detected between FXTp- group and normal controls (*p* = 0.99). To test if the group difference in the N400 repetition effect was mediated by the potential confounds of sex, *FMR1* mRNA level (normalized in relation to the mean value of the control group), or the CVLT measures of learning and memory, additional ANCOVA models were tested. These analyses showed that significant intergroup differences persisted after correcting for sex (*F* = 4.34, *p* = 0.02) or CVLT long delay cued recall (*F* = 4.40, *p* = 0.02), but narrowly missed statistical significance after correcting for CVLT learning of List A, trial 1–5 (*F* = 2.89, *p* = 0.075) or *FMR1* mRNA (*F* = 2.58, *p* = 0.10; data only available for *n* = 20).

No significant group differences were found for latency measures of N400 repetition effect.

#### Repetition of Congruous Words

**FIGURE 2:**
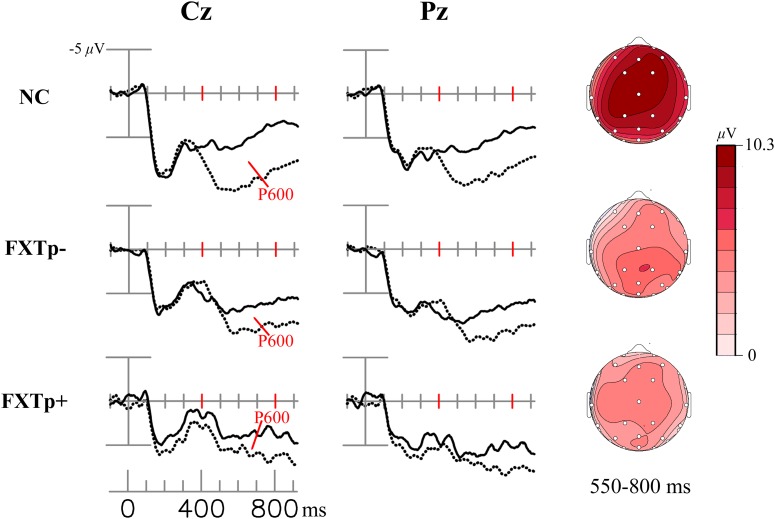
The LPC/P600 repetition effect. Columns to the left: grand average ERPs to initial (dotted line) and repeated (solid line) presentations of congruous new – old words at electrodes Cz (midline central) and Pz (midline parietal). Right column: topographical maps of the LCP/P600 repetition effect.

**Figure 2** shows word repetition effect of the LPC (i.e., the difference between ERPs to initial and repeated presentations of congruous target words).

Repeated measures ANOVA of the LPC mean amplitudes between 300 and 800 ms after the onset of target words demonstrated a significant main effect of *repetition* (*F* = 13.75, *p* = 0.0008, η^2^= 0.31), confirming the presence of an LPC repetition effect across all subjects. Despite the seemingly stronger positivities shown in the topographic maps of normal controls relative to patients, ANOVA of the LPC repetition effect amplitudes found no statistically significant group differences in the 300–550 ms time window (*F* = 1.4, *p* = 0.26) or in the 550–800 ms time window (*F* = 1.83, *p* = 0.18). No significant group differences were found for latency measures of LPC repetition effects.

#### N400 Congruity Effect to New Words

**FIGURE 3:**
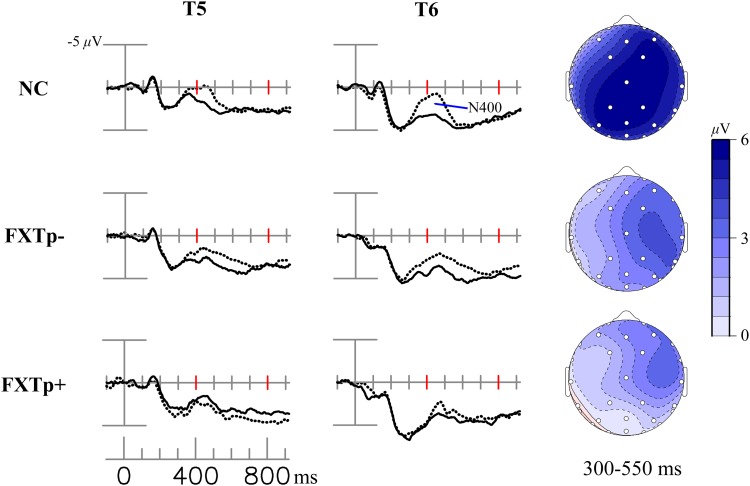
The N400 congruity effect. Columns to the left: grand average ERPs to incongruous initial (dotted line) and congruous initial (solid line) presentations of incongruous – congruous new words at posterior temporal electrodes T5 and T6. Right column: topographical maps of the N400 congruity effect.

**Figure 3** displays the N400 congruity effect (i.e., the difference between ERPs to initial presentations of congruous vs. incongruous target words).

Repeated measures ANOVA of the N400 mean amplitudes between 300 and 550 ms revealed a significant main effect of *congruity* across all participants (*F* = 22.12, *p* < 0.0001, η^2^= 0.42), confirming the presence of the N400 congruity effect. Though the topographic maps seem to visually show more prominent negativities in normal controls relative to patients, ANOVA of the N400 congruity effect amplitudes found no statistically significant differences among the three groups (*F* = 1.51, *p* = 0.24).

#### Correlational Results

Correlational tests revealed that increased N400 repetition effect amplitudes (averaged across the five right temporo-parietal electrodes Wr, T6, CP2, P4, and O2) were significantly correlated with higher scores on subsequent cued-recall (*r* = 0.61, *p* = 0.0002) and multiple choice recognition (*r* = 0.46, *p* = 0.009) for the congruous target words across all 33 participants (**Table 3**). To make positive correlation coefficients represent positive associations, N400 repetition effect used in correlational analysis was calculated by subtracting N400 to incongruous words from N400 to congruous words. Across all subjects, the association between larger N400 repetition effect amplitudes and higher verbal memory scores was also supported by the marginally significant correlations between N400 repetition effect and free recall scores for the congruous target words (*r* = 0.37, *p* = 0.04), learning/acquisition as measured by CVLT list A trials 1–5 (*r* = 0.44, *p* = 0.02), and CVLT long delay cued recall (*r* = 0.42, *p* = 0.03). Similar magnitude correlations were found within all 22 FXTAS patients (**Table 3**), but the correlations with CVLT measures both narrowly missed the threshold for statistical significance (*p*’s = 0.06 and 0.08). No correlations between N400 repetition effect and executive function, as measured by the BDS-2, were found. *FMR1* mRNA levels across all FXTAS patients (*n* = 14 had mRNA data) were inversely correlated with delayed recall memory as measured by CVLT (short delay free recall: *r* = -0.77, *p* = 0.002; short delay cued recall: *r* = -0.71, *p* = 0.006; long delay cued recall: *r* = -0.72, *p* = 0.005), but not with N400 repetition effect amplitude (*r* = 0.22, *p* = 0.44).

**Table 3:** Linear correlation coefficients between N400 repetition effect amplitude and neuropsychological test scores.

	All subjects		Two patient groups		FXTp-		FXTp+	
				
	*r*	*P*	*r*	*P*	*r*	*P*	*r*	*P*
BDS-2	0.14	0.47	0.29	0.23	-0.47	0.173	0.47	0.21
CVLT-long delay cued	0.42	0.03^∗^	0.40	0.08	0.28	0.41	0.66	0.054
CVLT list A, trials 1–5	0.44	0.02^∗^	0.42	0.06	-0.07	0.85	0.56	0.12
Free recall	0.37	0.04^∗^	0.56	0.01^∗^	0.78	0.0049^∗∗^	0.50	0.17
Cued recall	0.61	0.0002^∗∗∗^	0.69	0.0005^∗∗∗^	0.88	0.0003^∗∗∗^	0.55	0.10
Multiple choice	0.46	0.009^∗^	0.51	0.02^∗^	0.32	0.33	0.45	0.19


*The N400 repetition effect here is a composite score based on the mean amplitude across five posterior electrodes (Cp2, P4, Wr, T6, and O2). FXTp-, FXTAS patients without parkinsonism; FXTp+, FXTAS patients with parkinsonism. ^∗^P < 0.05; ^∗∗^P < 0.005; ^∗∗∗^P < 0.001.*

## Discussion

Although parkinsonian features are common in FXTAS and represent a minor diagnostic criterion for FXTAS, characteristics and trajectories of cognitive functioning in a subtype of FXTAS with parkinsonism (defined as having a FXTAS diagnosis along with bradykinesia and at least one other main parkinsonian feature) remains largely understudied. The current study examined cognitive abilities in FXTAS patients with (FXTp+) and without parkinsonism (FXTp-) using an ERP word repetition experiment and neuropsychological testing. The results showed that, when compared with FXTp- and NC, the FXTp+ group demonstrated more severe deficits in executive function and verbal memory, along with a significantly reduced N400 repetition effect which was correlated with verbal memory both for target words used in the ERP experiment and on CVLT. Across all FXTAS patients, *FMR1* mRNA levels were inversely correlated with delayed recalls on CVLT. This study represents the first to characterize both cognitive and electrophysiological profiles in FXTAS with and without parkinsonism.

Executive dysfunction and verbal learning/acquisition deterioration have been shown to be the primary cognitive deficits in PD patients without dementia. Muslimovic et al. (2005) examined 115 newly diagnosed PD patients and found that cognitive impairments in these patients are mediated by immediate memory and executive function, and that the largest effect size was found on immediate verbal memory on the Rey Auditory Verbal Learning Test (RAVLT; trials 1–5). (Muslimovic et al., 2005) Aarsland et al. (2009) investigated 196 drug-naïve PD patients and obtained largest effect size for verbal memory (both immediate and delayed) on CVLT-2. The present findings of more prominent executive dysfunction and verbal memory deficits in FXTp+ than FXTp- are thus consistent with those in PD patients, and may indicate that concomitant and/or synergistic pathogenetic mechanisms associated with PD play a role in neurodegeneration among patients with FXTAS. As mentioned above, studies in carriers of slightly expanded CGG repeat *FMR1* alleles with a diagnosis of PD have observed more severe cognitive deficits in general ability, memory, and attention domains when compared with their disease controls with PD (Loesch et al., 2011; Trost et al., 2014). Although there has been controversy regarding the neural substrates of executive function, lesion data and neuroimaging studies generally agree that, broadly speaking, prefrontal cortex is the key region supporting executive function. Interestingly, decreased functional connectivities between right prefrontal regions and right hippocampus were observed in a group of male premutation carriers without FXTAS during a working memory task (Wang et al., 2012).

The N400 effect is a well-established electrophysiological index of semantic priming and semantic processing load. Yang et al. (2014a) have found that the N400 repetition effect amplitude is the strongest predictor of the CVLT short delay cued recall score in patients with FXTAS and was correlated significantly with all 6 CVLT measures of verbal learning and memory in the FXTAS group, but not in a group with prodromal/early AD. These authors suggested that the N400 repetition effect, shown to primarily originate from temporal neocortex (Nobre et al., 1994; Halgren et al., 2002), has substantial association with learning/acquisition and short delayed recall, and is sensitive to memory dysfunction in FXTAS.

The pathophysiological mechanisms underlying parkinsonism in FXTAS remains elusive. Loesch et al. (2011) found reduced dehydrogenase subunit 1 mitochondrial gene in whole blood in carriers compared to disease controls with typical and atypical PD. The authors thus suggested the involvement of mitochondrial dysfunction in the development of parkinsonism in carriers of the *FMR1* gray-zone and premutation alleles. Hagerman and Hagerman (2016) indicated that the pathological development of PD in FXTAS might also be facilitated by extracellular deposition of iron. Another pathogenic mechanism to consider is the proteasomal degradation pathway that was found to be affected in both FXTAS and PD (Moore et al., 2003; Iwahashi et al., 2006). Finally, studies have found that several autopsied cases with FXTAS also had superimposed α-synuclein containing Lewy body pathology (Greco et al., 2002; De Pablo-Fernandez et al., 2015). How these possible mechanisms contribute to the cognitive and electrophysiological phenotypes observed in FXTp+ require substantial further investigations.

## Conclusion

To conclude, when compared to both normal elderly controls and FXTAS patients without parkinsonism, FXTAS patients with parkinsonism present more severe deficits in executive function and verbal learning, as well as a significantly reduced N400 repetition effect which was inversely correlated with verbal learning and verbal memory abilities. It has been reported that, in the first randomized controlled clinical trial for FXTAS, the N400 ERP repetition effect was improved after 12 months of treatment with the uncompetitive NMDA antagonist memantine, and the N400 improvement was correlated with better cued-recall of the experimental verbal stimuli (Yang et al., 2014b). Therefore, the current findings have implications not only for understanding the cognitive impairments associated with the parkinsonism subtype of FXTAS, but also for the development and validation of new pharmaceutical (e.g., memantine in combination with L-dopa) and behavioral intervention for these cognitive impairments. Study limitations include limited sample sizes, incomplete data of some measures (e.g., *FMR1* mRNA level), and lack of neurophysiological confirmation of clinical diagnosis. Replication of these results in independent samples will be important as will be further studies to characterize cognition, biomarkers, and neuropathology in larger samples of FXTAS patients with Parkinsonism.

## Author Contributions

X-HW, J-CY, LZ, and JO conceived and designed the study. X-HW, J-CY, RS, DC, MY, JD, Y-QN, FT, JX, and JO acquired and analyzed the data. X-HW, J-CY, RS, DC, SY, FT, RH, LZ, JX, and JO drafted and revised the manuscript.

## Conflict of Interest Statement

RH has received funding from Roche, Novartis, Forest, Seaside Therapeutics, and Curemark for clinical trials in fragile X syndrome or autism. She has consulted with Novartis, Zynerba, and Ovid regarding clinical trials in fragile X syndrome. JO has received support from Genentech and Eli-Lilly for clinical drug trials, and has served as a consultant for Lundbeck Pharmaceuticals. The remaining authors declare that the research was conducted in the absence of any commercial or financial relationships that could be construed as a potential conflict of interest.
